# Schwann Cells Promote Myogenic Differentiation of Myoblasts and Adipogenic Mesenchymal Stromal Cells on Poly-ɛ-Caprolactone-Collagen I-Nanofibers

**DOI:** 10.3390/cells11091436

**Published:** 2022-04-24

**Authors:** Aijia Cai, Zeng-Ming Zheng, Marcus Himmler, Dirk W. Schubert, Thomas A. Fuchsluger, Volker Weisbach, Raymund E. Horch, Andreas Arkudas

**Affiliations:** 1 Laboratory for Tissue Engineering and Regenerative Medicine, Department of Plastic and Hand Surgery, University Hospital of Erlangen, Friedrich-Alexander-University Erlangen-Nürnberg (FAU), 91054 Erlangen, Germany; zhengzengming@163.com (Z.-M.Z.); raymund.horch@uk-erlangen.de (R.E.H.); andreas.arkudas@uk-erlangen.de (A.A.); 2 Institute of Polymer Materials, Department of Materials Science and Engineering, University of Erlangen-Nürnberg (FAU), Martensstrasse 7, 91058 Erlangen, Germany; marcus.himmler@fau.de (M.H.); dirk.schubert@fau.de (D.W.S.); 3 Department of Ophthalmology, University Medical Center Rostock, Doberaner Str. 140, 18057 Rostock, Germany; thomas.fuchsluger@med.uni-rostock.de; 4 Department of Transfusion Medicine, University Hospital of Erlangen, Friedrich-Alexander-University Erlangen-Nürnberg (FAU), 91054 Erlangen, Germany; volker.weisbach@uk-erlangen.de

**Keywords:** ADSC, myoblasts, myogenic differentiation, mesenchymal stem cells, nanofibers, Schwann cells

## Abstract

For the purpose of skeletal muscle tissue engineering, different cell types have been investigated regarding their myogenic differentiation potential, including co-cultured myoblasts and adipogenic mesenchymal stromal cells (Mb/ADSC). As neural cells enhance synaptic junction formation, the aim of this study was to co-culture Schwann cells (SCs) with Mb/ADSC on biocompatible electrospun aligned poly-ε-polycaprolacton (PCL)-collagen I-nanofibers. It was hypothesized that SCs, as part of the peripheral nervous system, promote the myogenic differentiation of Mb/ADSC co-cultures. Mb/ADSC were compared to Mb/ADSC/SC regarding their capacity for myogenic differentiation via immunofluorescent staining and gene expression of myogenic markers. Mb/ADSC/SC showed more myotubes after 28 days of differentiation (*p* ≤ 0.05). After 28 days of differentiation on electrospun aligned PCL-collagen I-nanofibers, gene expression of myosin heavy chains (MYH2) and myogenin (MYOG) was upregulated in Mb/ADSC/SC compared to Mb/ADSC (*p* ≤ 0.01 and *p* ≤ 0.05, respectively). Immunofluorescent staining for MHC showed highly aligned multinucleated cells as possible myotube formation in Mb/ADSC/SC. In conclusion, SCs promote myogenic differentiation of Mb/ADSC. The co-culture of primary Mb/ADSC/SC on PCL-collagen I-nanofibers serves as a physiological model for skeletal muscle tissue engineering, applicable to future clinical applications.

## 1. Introduction

Volumetric muscle loss exceeds the natural regeneration capacity of skeletal muscle tissue and depends on the transfer of healthy donor tissue, including free autologous muscle flaps if available. Since this comes along with donor site morbidity, high hopes have been put into skeletal muscle tissue engineering, which might help avoid sacrificing substantial functional muscle tissue [1,2,3,4]. The creation of transplantable and functional three-dimensional (3D) muscle tissue necessitates cells with myogenic capacity, an adequate platform, vascularization for large volume constructs, and innervation. An ideal cell candidate is the myoblast (Mb), a muscle progenitor cell that plays an important role in muscle regeneration. During myogenesis, activated Mbs commit to differentiation and fuse into multinucleated myotubes [5]. While primary Mbs lose their myogenic differentiation capacity during high passages, mesenchymal stem cells (MSCs) can be expanded to high passages without losing their ability to differentiate into different tissues [6]. Compared to other types of MSC, adipogenic mesenchymal stromal cells (ADSCs) can be easily harvested from fat tissue in abundant quantities and by minimally invasive procedures [7]. Their regenerative potential has been proven in various studies [8,9].

In terms of axially oriented skeletal muscle tissue, highly aligned electrospun nanofibers were suggested as a promising 3D matrix [10,11]. In a prior study, co-cultured ADSCs and Mbs were myogenically differentiated on electrospun poly-ε-polycaprolacton (PCL)-collagen I-nanofibers [12]. The aligned nanofibers not only mimicked the structure of myofibers in skeletal muscle but also showed stability and biocompatibility during long-term myogenic differentiation of co-cultured Mbs and MSCs [12,13].

Another important aspect of skeletal muscle tissue engineering is the creation of functional contractible tissue. For this purpose, the use of neuronal cells could be a solution for functionalizing the tissue and enhancing myogenic differentiation [14]. Multiple studies have shown the formation of neuromuscular junctions with subsequent myotube maturation and neurotization by the addition of neuronal progenitor cells like embryonal motor neurons or neuronal slices from newborn rats [15,16]. Unfortunately, those cell types limit the study design to be solely of an in vitro nature, for example as part of a pharmacological study [15]. Another type of neural cell are the Schwann cells (SC), which form myelin sheaths around peripheral nerve axons and interact with motoneurons to form neuromuscular junctions. This cell type modulates synaptic activity and regenerates neuromuscular junctions after nerve injury [17]. As peripheral nerve injury leads to muscle atrophy and dysfunction, SCs could play a role in regenerating muscle tissue. It has been shown that both nerve and muscle regeneration are regulated via the same pathways like the Wnt/β-catenin [18] or the JAK2/STAT3 signaling pathway [19]. There have been tri-culture models of motoneurons, Mbs, and SCs [20,21]. In those studies, the addition of SCs led to enhanced myotube formation [20,21] and increased viability of the myotubes [20]. However, similarly to the above mentioned studies, those models utilized cell lines or human pluripotent induced stem cells, which again makes their use for clinical translation unsuitable due to possible tumor formation [22,23]. For regenerative purposes, the co-culture of primary cells would be more appropriate.

In order to determine whether SCs promote myogenic differentiation, we set out a study, comparing Mb/ADSC to Mb/ADSC/SC co-cultured on PCL-collagen I-nanofibers. The use of primary cells on a biocompatible matrix was supposed to facilitate later clinical application. It was hypothesized that the addition of SCs enhances myogenic differentiation of Mb/ADSC co-cultures.

## 2. Materials and Methods

### 2.1. Cell Isolation and Culture

Tissue collection was approved by the institutional ethics committee (approval number 424_18 B). Informed consent was obtained from all patients. Primary human Mbs were isolated from three patients undergoing free muscle flaps as described previously [12,24]. Briefly, muscle tissue was digested with collagenase (CLS2, Biochrom GmbH, Berlin, Germany), Dispase^®^ II (Sigma Aldrich, St. Louis, MO, USA), and trypsin (Sigma Aldrich). Cells were plated into type I collagen-coated flasks (rat tail collagen, Sigma Aldrich). As part of a pre-plate technique for the enrichment of Mbs, the supernatant containing non-adherent cells was collected and re-plated in a new coated flask after two hours. This step was repeated every 24 h until the third pre-plated cells (PP3) were further passaged. PP3 Mbss of passage 4 (P4) showed approximately 95% desmin positive cells in immunofluorescent images (ab8470, Abcam, Cambridge, UK). Human primary fibroblasts (HFIB-D, cryo, provitro AG, Berlin, Germany) in P9 served as a negative control. To verify myotube formation capacity, Mbs of passage 5 (P5) were stained for desmin (ab8470, Abcam) after 7 days of myogenic differentiation induced by myogenic differentiation medium containing DMEM/Ham’s F12 (Gibco, Carlsbad, CA, USA) + 2% donor horse serum (DHS, Biochrom GmbH) + 1% L-glutamine + 1% penicillin/streptomycin (P/S) (Biochrom GmbH) + 0.4 µg/mL dexamethasone (Sigma Aldrich), 1 ng/mL basic fibroblast growth factor (bFGF) (Peprotech, Hamburg, Germany).

Human ADSCs were isolated from one patient undergoing abdominoplasty as previously described [25]. Cells were allowed to differentiate into chondrocytes, adipocytes, and osteocytes with specific differentiation media (Pelobiotech GmbH, Planegg, Germany). Flow cytometry was performed to characterize ADSCs in passage 3 (P3) and passage 6 (P6). The following fluorescent conjugated antibodies were used: CD90, CD73, CD105, SSEA-4, CD271, CD31, and Lin as a cocktail of negative markers including CD34, CD11b, CD19, CD45, HLA-DR (BD Biosciences, Heidelberg, Germany). Detection of fluorochrome labeling was performed on a fluorescence activated cell sorting cytometer (FACSCalibur, Becton Dickinson, NJ, USA) with FACScan^®^ with cell quest^®^ software (Becton Dickinson).

Human Schwann cells (SCs) were purchased from Innoprot (P10351, Derio, Spain) and were cultured as recommended by the manufacturer. SCs in P5 were immunostained for GFAP (glial fibrillary acidic protein, ASTR06, Neomarkers, Portsmouth, NH, USA), S100 (ab76749, Abcam), and p75 (G323A, Promega, Madison, WI, USA). Myoblasts served as a negative control.

For each experiment, myoblasts from the three different donors (each in P5, n = 3) were co-cultured with ADSCs (in P6) alone in a ratio of 1:1 (Mb/ADSC) or in combination with SCs (in P5) (Mb/ADSC/SC).

To determine the ideal concentration of Mbs, ADSCs, and SCs, three groups were formed with different ratios of Mb/ADSC/SC (1:1:1; 1:1:0.5; 1:1:0.25) and co-cultured two-dimensionally (2D) in 6-well culture plates at a density of 300,000 cells. Creatine kinase (CK) assay (Abcam) was performed, as described previously, after 3, 7, and 14 days of myogenic differentiation [12]. Briefly, creatine and adenosine triphosphate were enzymatically converted into phosphocreatine and adenosine diphosphate (ADP) after cells were resuspended in a CK assay buffer. The addition of reaction mix induced the generation of nicotinamide adenine dinucleotide (NADH), which was measured photometrically at 450 nm. Based on those results, a ratio of 1:1:0.5 was used for Mb/ADSC/SC for further experiments.

### 2.2. 2D Myogenic Differentiation of Mb/ADSC and Mb/ADSC/SC

Mb/ADSC (1:1) and Mb/ADSC/SC (1:1:0.5) were co-cultured in 24-well culture plates at a density of 20,000 cells. Myogenic differentiation was initiated when cells reached confluence and continued for 7, 14, and 28 days. After each time period, cells were fixed and stained for fast myosin skeletal heavy chain (MHC) (ab91506, Abcam) as described before [12]. Pictures were taken of each quadrant of every well in 10× magnification with a fluorescent microscope (IX83, cellSens, software, Olympus, Hamburg, Germany). The myotube fusion index (MFI) was calculated as the ratio of the nuclei number in myotubes with two or more nuclei vs. the total number of nuclei, whereas the myotube maturation index (MMI) was defined as percent of myotubes with five or more nuclei [26]. Images were quantified with ImageJ 1.53e (National Institutes of Health, Bethesda, MD, USA). The number of total nuclei was assessed by splitting color channels and by inverting the blue channel, representing the DAPI-stained nuclei. After adjusting threshold and applying watershed, particles were analyzed, revealing the total number of nuclei automatically. The multi-point tool was used for counting nuclei in myotubes manually. The analysis of MFI and MMI was performed by a blinded investigator (A. C.).

### 2.3. Electrospinning of PCL-Collagen I-Nanofibers and 3D Cell Seeding

Aligned PCL-collagen I-nanofibers were electrospun as described previously [12,13,27]. Acetic acid (Carl Roth GmbH, Karlsruhe, Germany) in a concentration of 90% was used as a benign solvent. Scanning electron microscopy (SEM) images were taken with an Auriga Fib-scanning electron microscope (SEM) (Zeiss, Oberkochen, Germany) after scaffolds were sputter-coated with gold using a Q150T Turbo-pumped Sputter Coater (Quorum Technologies Inc., Guelph, ON, Canada).

For 3D cultivation, Mb/ADSC or Mb/ADSC/SC were seeded at a density of 300,000 cells on PCL-collagen I-nanoscaffolds. For each experiment, three scaffolds were used per group (one Mb isolation per scaffold, n = 3). Cells were allowed to proliferate for 7 days before differentiation was started [12]. Myogenic differentiation was continued for a period of 28 days. Thereafter, cells were collected for RNA isolation or seeded scaffolds were fixed for immunofluorescent staining. Wst-8 and live dead assay were obtained after 0, 14, and 28 days of myogenic differentiation.

### 2.4. RNA Isolation and Quantitative PCR Analysis

Gene expression of MYH2 (myosin heavy chain 2), ACTA1 (skeletal alpha actin), and MYOG (myogenin) was analyzed in 3D co-cultures. GAPDH (glyceraldehyde-3-phosphate dehydrogenase) was used as housekeeping gene and RNA from human muscle tissue served as positive control. Scaffolds as well as human muscle were digested in Trizol (Life Technologies, Carlsbad, CA, USA) and chloroform [10] to extract RNA, which was reverse-transcribed into cDNA with a QuantiTect Reverse Transcription Kit and a Sensiscript Reverse Transcription Kit (both from Qiagen GmbH). Triplicets of cDNA were amplified through quantitative real-time PCR using SsoAdvanced Universal SYBR Green PCR Supermix (Bio-Rad, Hercules, CA, USA) and a Light Cycler (Bio-Rad CFX96 Touch^TM^). Relative gene expression was calculated using the 2^−ΔΔCt^ method. The primer sequences used are listed in Table 1.

### 2.5. Cell Viability on PCL-Collagen I-Nanoscaffolds

3D co-cultures were allowed to proliferate for 7 days before myogenic differentiation was induced for 0, 14, and 28 days. Water-soluble tetrazolium salt (Wst)-8-assay (Promokine, Promocell GmbH, Heidelberg, Germany) of the seeded scaffolds was performed as triplicates as described previously [12]. For assessment of cell viability, absorbance was measured at 450 nm with Photometer Thermo Scientific™ Multiskan™ GO. Afterwards, live dead assay (apoptotoc/necrotic/healthy cells detection kit, PromoKine, Heidelberg, Germany) was performed according to the manufacturer’s instructions. 10× magnified images of every quadrant of each scaffold were taken with a fluorescence microscope (IX83, cellSens, software, Olympus). ImageJ 1.53e (National Institutes of Health, Bethesda, MD, USA) was used to quantify live, apoptotic, and necrotic cells by splitting color channels and inverting blue (live cells), red (necrotic cells), and green (apoptotic cells). An automatic cell count was carried out of each channel as described above. Necrotic index (NI) and apoptotic index (AI) were calculated as number of red or green cells in relation to total cell number, respectively.

### 2.6. Immunofluorescence

Mbs of PP3 in P4 were seeded on collagen-coated 48-well plates at a density of 5000 cells. After 24 h, cells were fixed, blocked, and incubated with desmin primary antibody (ab8470, Abcam) at 0.5 µg/mL for one hour. Mbs in P5 were myogenically differentiated for 7 days and fixed for desmin staining as previously described.

SCs in P5 were seeded on 48-well plates at a density of 5000 cells and fixed after 24 h. Cells were stained with GFAP primary antibody (ASTR06, Neomarkers) at 20 µg/mL, S100 primary antibody (ab76749, Abcam) at 4 µg/mL, and p75 antibody (G323A, Promega) at 20 µg/mL for 2 h at room temperature. Myoblasts served as a negative control.

Mb/ADSC or Mb/ADSC/SC were seeded in an expansion medium at a density of 300,000 cells on PCL-collagen I-nanoscaffolds. After 7 days, myogenic differentiation was continued for 4 weeks. Scaffolds were covered with anti-fast MHC antibody (ab91506, Abcam) at 5 µg/mL for 1 h at room temperature.

Alexa Fluor 647 goat anti-rabbit IgG H&L (ab150083, Abcam) was used as secondary antibody at 4 µg/mL for 30 min at room temperature for all primary antibodies except for GFAP, which was visualized with Alexa Fluor 594 goat anti-mouse (A21121, Life Technologies). Probes were counterstained with 1 µg/mL diamidine-phenylindole-dihydrochloride (DAPI, Thermofisher Scientific Inc., Waltham, MA, USA) for 5 min before cells were photographed with a fluorescence microscope (IX83, cellSens, software, Olympus).

### 2.7. Statistical Analysis

Data are expressed as the mean with standard deviations. Data normality was tested with Shapiro-Wilk test. One-way analysis of variance (ANOVA) with Tukey’s correction or Friedman test with Dunn’s correction for multiple comparisons were used, as appropriate. Paired comparisons at different time points were carried out using repeated measures ANOVA with Tukey’s multiple comparisons test for post hoc analysis. Pairwise comparison between Mb/ADSC and Mb/ADSC/SC was carried out using unpaired *t*-tests or Mann-Whitney tests, as appropriate. Statistical analysis was performed using GraphPad Prism version 8 (La Jolla, CA, USA). A *p*-value ≤ 0.05 was considered statistically significant.

## 3. Results

### 3.1. Myoblast, ADSC, and Schwann Cell Characterization

**Figure 1:**
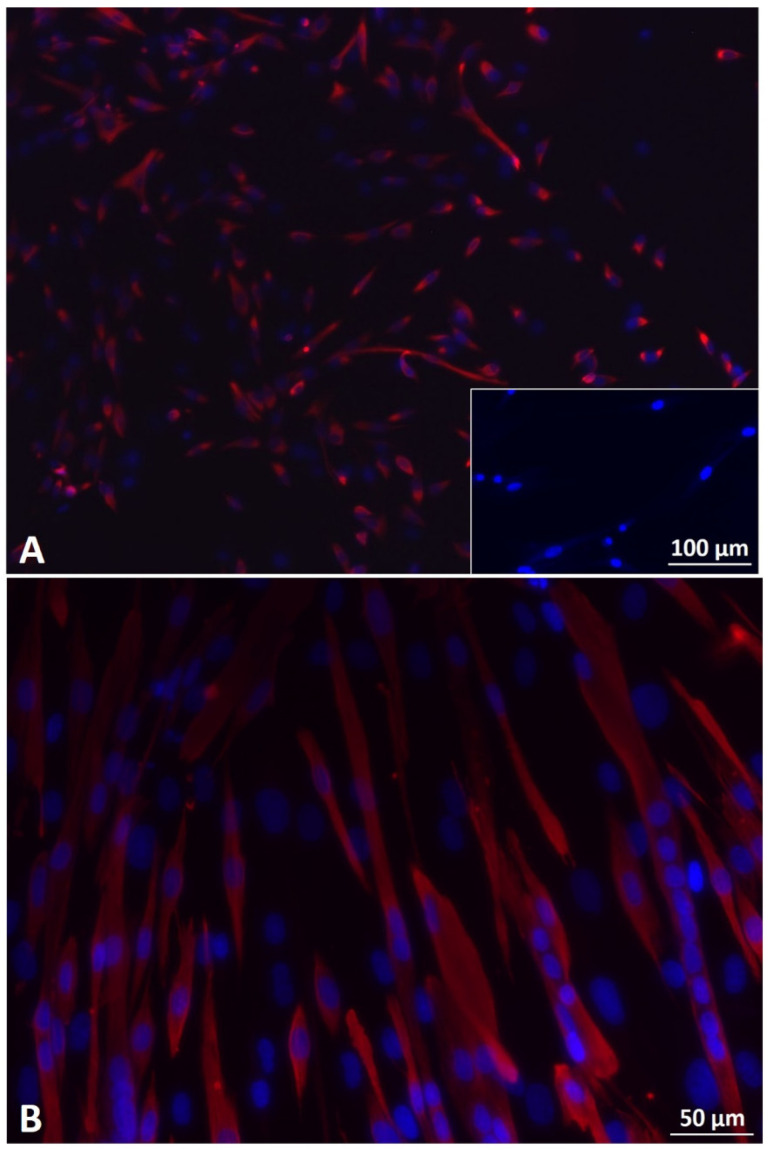
(**A**) Fluorescence microscopy of desmin-positive (red) human primary myoblasts (Mb) in pre-plate 3 (PP3) and passage 4 (P4). Merge with DAPI (blue) shows that nearly all cells were desmin-positive. Insert shows human primary fibroblasts in P9 negative for desmin. Scale bar 100 µm applies to insert as well as to main image. (**B**) Fluorescence microscopy of Mbs in PP3 and P5 after seven days of myogenic differentiation. Merge with DAPI (blue) shows that cells were desmin-positive (red) and formed multinucleated cells, indicating myotube formation. Scale bar 50 µm.

**Figure 2:**
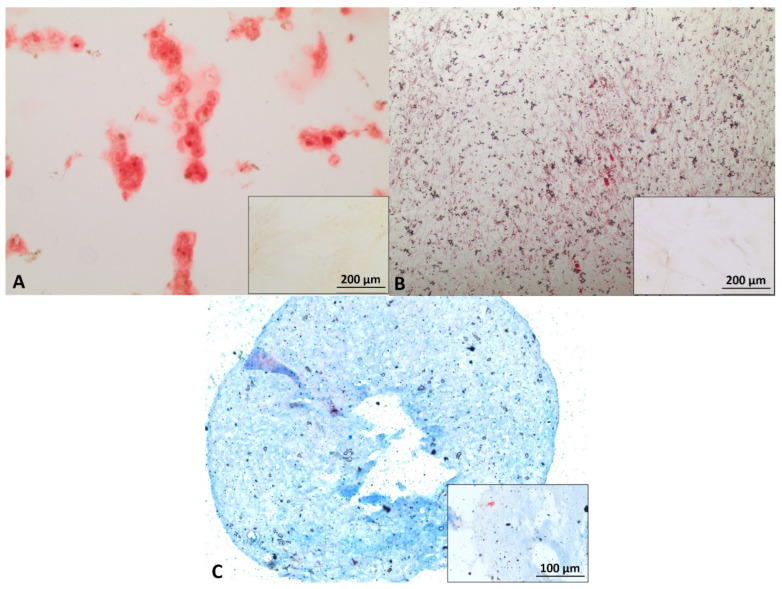
Characterization of human adipose derived stem cells (ADSC). ADSCs in P4 were differentiated into osteocytes (**A**), adipocytes (**B**), and chondrocytes (**C**). Calcium deposits were stained with Alizarin Red S (**A**). Lipid vacuoles were visualized with oil red O staining (**B**). Proteoglycans of chondrogenic pellets were detected with Alcian blue staining (**C**). Inserts represent ADSC, cultured in proliferation medium as negative controls. Scale bars 200 µm (**A**,**B**) and 100 µm (**C**) refer to inserts as well as to main images.

**Figure 3:**
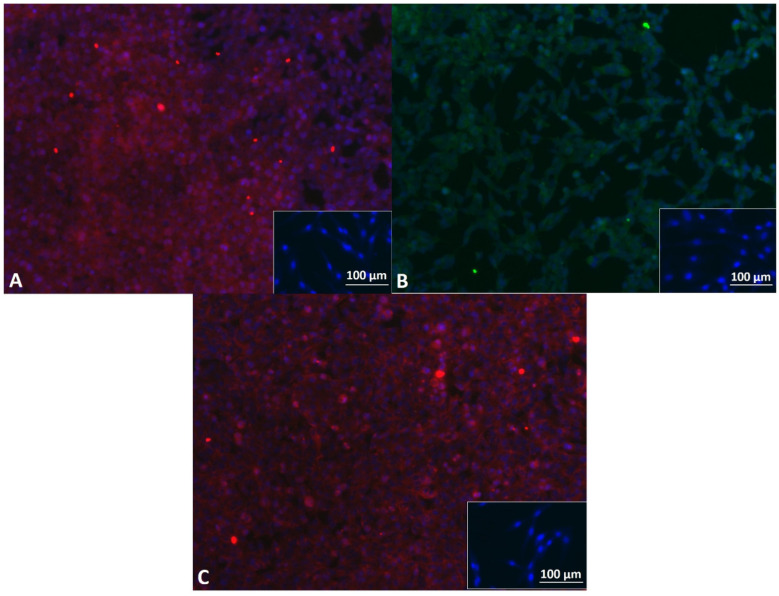
Fluorescence microscopy of human Schwann cells (SC) in P5. Cells were positive for glial fibrillary acidic protein (GFAP) (red) (**A**), S100 (green) (**B**), and p75 (red) (**C**). Inserts represent human Mbs (blue = DAPI) as negative controls. Scale bars 100 µm apply to inserts as well as to main images.

Mbs in PP3 of P4 showed approximately 95% desmin positive cells. Mbs in PP3 of P5 were still desmin positive and showed myotube formation after 7 days of myogenic differentiation (Figure 1A,B). Figure S1 (Supplementary Material) shows characterization of Mbs in detail. ADSCs were successfully differentiated into chondrogenic, osteogenic, and adipogenic lineages (Figure 2). They were analyzed for cell surface markers in low (P3) and high passages (P6). Cells were positive for CD90 (100% in both passages), CD73 (100% in both passages), and CD105 (99.3% and 99.6%, respectively) (Figure S2, Supplementary Material). SCs in P5 were positive for GFAP, S100, and p75, each representing characteristic SC markers, while myoblasts were negative (Figure 3).

### 3.2. Optimal Cell Ratio of Mb/ADSC/SC

**Figure 4:**
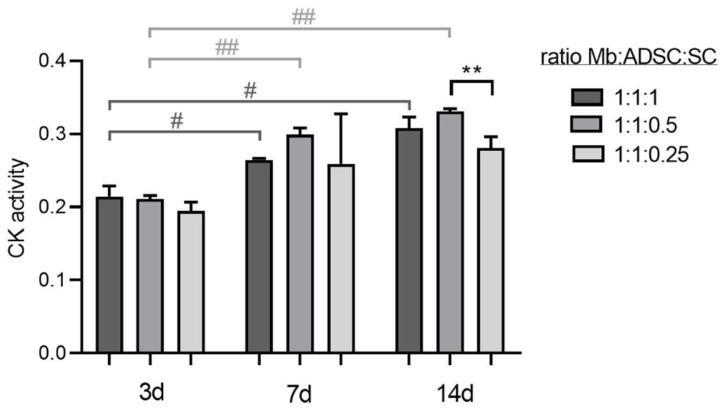
Optimal ratio of Mb/ADSC/SC determined with creatine kinase (CK) activity. Values are presented in mean ± standard deviation. CK activity increased over time within 1:1:1 (# *p* < 0.05, repeated measures ANOVA with Tukey’s correction for multiple comparisons) and 1:1:0.5 (## *p* < 0.01, repeated measures ANOVA with Tukey’s correction for multiple comparisons). After 14 days, CK activity was lower in 1:1:0.25 groups compared to 1:1:0.5 groups (** *p* < 0.01, one way ANOVA with Tukey’s correction for multiple comparisons). Based on those results, Mb/ADSC/SC were used at a ratio of 1:1:0.5 for further experiments.

Mb/ADSC/SC were 2D co-cultured in different ratios (1:1:1, 1:1:0.5, 1:1:0.25) and myogenically differentiated for different time periods. CK activity as an indicator for myogenic differentiation increased over time within 1:1:1 (*p* = 0.035 and *p* = 0.018 from 3 to 7 and 3 to 14 days, respectively) and 1:1:0.5 (*p* = 0.007 and *p* = 0.001 from 3 to 7 and 3 to 14 days, respectively). After 3 and 7 days of myogenic differentiation, CK activity did not differ between groups (3 days: *p* = 0.929 between 1:1:1 and 1:1:0.5, *p* = 0.152 between 1:1:1 and 1:1:0.25, *p* = 0.241 between 1:1:0.5 and 1:1:0.25; 7 days: *p* = 0.569 between 1:1:1 and 1:1:0.5, *p* = 0.985 between 1:1:1 and 1:1:0.25, *p* = 0.482 between 1:1:0.5 and 1:1:0.25). After 14 days, comparisons between different ratio groups showed a decrease of CK activity in 1:1:0.25 groups compared to 1:1:0.5 groups (*p* = 0.006) (Figure 4). Based on these results, Mb/ADSC/SC were used at a ratio of 1:1:0.5 for further experiments.

### 3.3. 2D Myogenic Differentiation of Mb/ADSC vs. Mb/ADSC/SC

**Figure 5:**
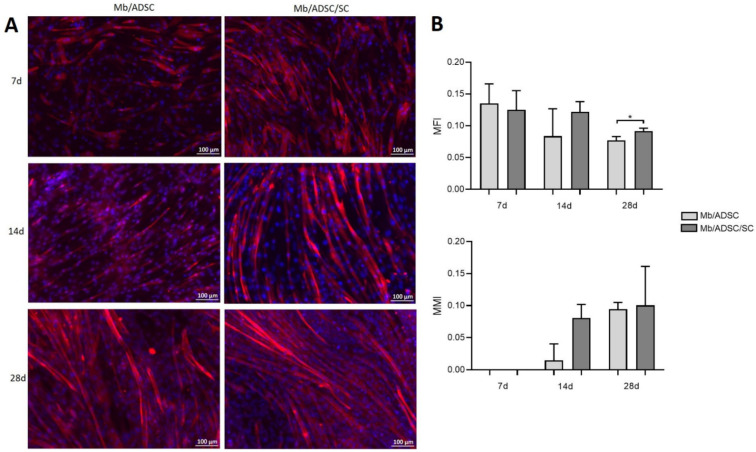
Myotube formation after 2D myogenic differentiation of Mb/ADSC vs. Mb/ADSC/SC. Values are presented in mean ± standard deviation. (**A**) Fluorescence microscopy of 2D co-cultures shows myosine heavy chain (MHC) positive (red) multinucleated (DAPI = blue) cells after 7, 14, and 28 days of myogenic differentiation. Scale bar 100 µm. (**B**) Myotube fusion index (MFI = nuclei in myotubes/total nuclei) was higher in Mb/ADSC/SC after 28 days (* *p* < 0.05, unpaired *t*-test). Myotube maturation index (MMI = myotubes with five or more nuclei/total myotubes) did not differ between Mb/ADSC and Mb/ADSC/SC during all time periods.

2D co-cultures were myogenically differentiated and immunostained for MHC (Figure 5A). MFI did not differ between Mb/ADSC and Mb/ADSC/SC after 7 or 14 days (*p* = 0.704 and *p* = 0.700, respectively). After 28 days of myogenic differentiation, Mb/ADSC/SC showed more myotubes than Mb/ADSC (*p* = 0.031). There were no maturated myotubes (myotubes with 5 or more nuclei) after 7 days. There was a tendency towards higher MMI for Mb/ADSC/SC compared to Mb/ADSC after 14 days (*p* = 0.0765) though not statistically significant. After 28 days, MMI was the same in both groups (*p* = 0.876) (Figure 5B).

### 3.4. Cell Viability on PCL-Collagen I-Nanoscaffolds

**Figure 6:**
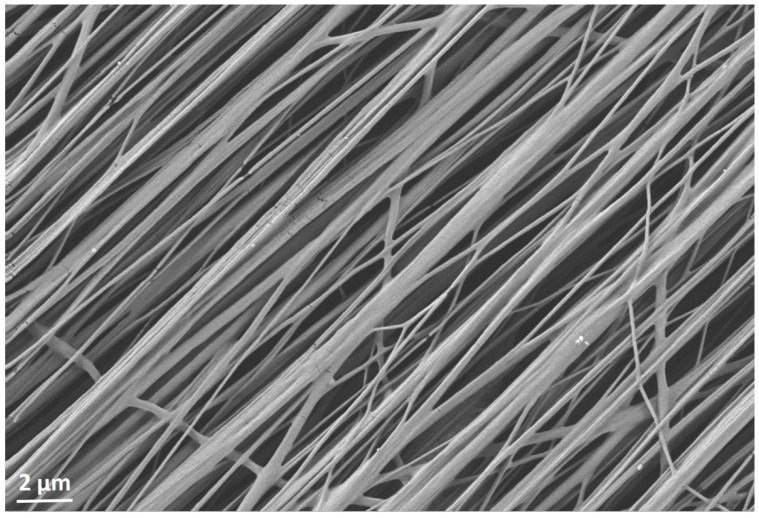
SEM image of electrospun aligned PCL-collagen I-nanofibers. Scale bar 2 µm.

**Figure 7:**
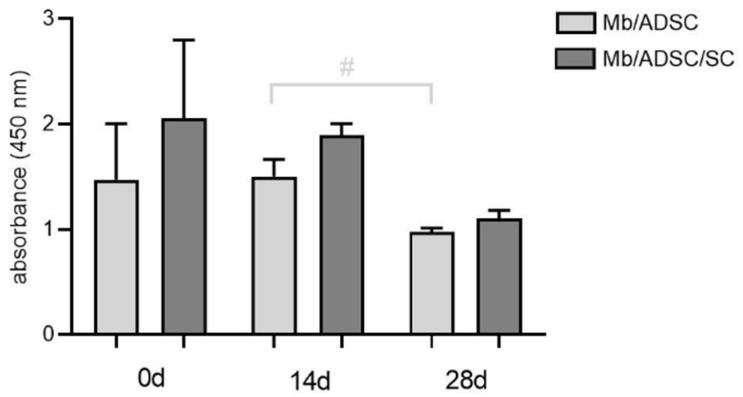
Cell viability on PCL-collagen I-nanofibers determined with wst-8-assay. Absorbance at a wave length of 450 nm is expressed as mean ± standard deviation. Cell viability decreased from 14 to 28 days in Mb/ADSC (# *p* < 0.05, repeated measures ANOVA with Tukey‘s multiple comparison test).

**Figure 8:**
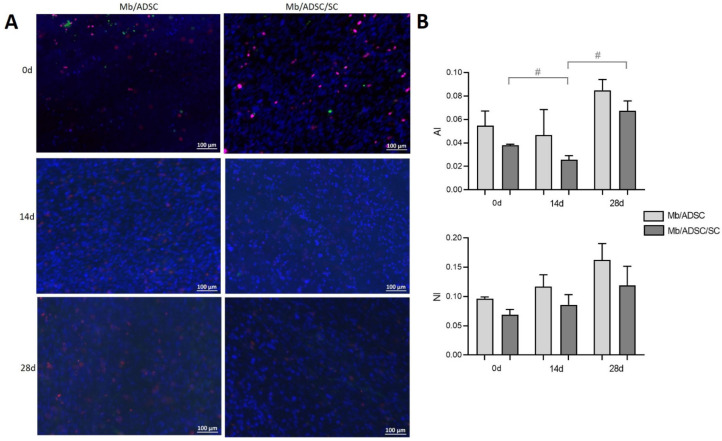
Cell status on PCL-collagen I-nanofibers determined with live dead assay. (**A**) After 0, 14, and 28 days of myogenic differentiation, live cells are demonstrated in blue, apoptotic cells in green, and necrotic cells in red. Scale bar 100 µm. (**B**) Apoptotic index (AI) and necrotic index (NI) did not differ between Mb/ADSC and Mb/ADSC/SC (unpaired *t*-test). AI decreased from 0 days to 14 days (# *p* < 0.05, repeated measures ANOVA with Tukey’s correction for multiple comparisons) and increased from 14 days to 28 days (# *p* < 0.05, repeated measures ANOVA with Tukey’s correction for multiple comparisons) for Mb/ADSC/SC.

3D co-cultures of Mb/ADSC or Mb/ADSC/SC were performed on PCL-collagen I-nanoscaffolds. SEM analysis showed parallel alignment of nanofibers (Figure 6) After 7 days of proliferation, myogenic differentiation was induced and continued for 14 and 28 days. After each time period (0, 14, and 28 days of differentiation), cell viability was assessed (Figure 7). While cell viability did not differ between different time periods for Mb/ADSC/SC (*p* = 1.000 between 0 and 14 days, *p* = 0.307 between 0 and 28 days, *p* = 0.124 between 14 and 28 days), it decreased from 14 days to 28 days for Mb/ADSC (*p* = 0.046). There was no difference concerning cell viability between Mb/ADSC and Mb/ADSC/SC during each time period although there was a tendency towards higher cell viability for Mb/ADSC/SC compared to Mb/ADSC after 28 days (*p* = 0.333 after 0 days, *p* = 0.100 after 14 days, *p* = 0.068 after 28 days). Live dead assay demonstrated live, apoptotic, and necrotic cells (Figure 8A). There was a tendency towards higher AI and NI for Mb/ADSC compared to Mb/ADSC/SC after 0 days of myogenic differentiation on PCL-collagen I-nanoscaffolds (*p* = 0.084, *p* = 0.077, respectively). After 14 days of myogenic differentiation, both AI and NI did not differ between groups (*p* = 0.175 and *p* = 0.112, respectively) as did NI after 28 days (*p* = 0.157) while there was a tendency towards higher AI for Mb/ADSC after 28 days (*p* = 0.071). While NI did not change within groups over time, AI decreased from day 0 to day 14 (*p* = 0.024) and increased from day 14 to day 28 (*p* = 0.048) for Mb/ADSC/SC (Figure 8B).

### 3.5. Myogenic Differentiation on PCL-Collagen I-Nanoscaffolds

**Figure 9:**
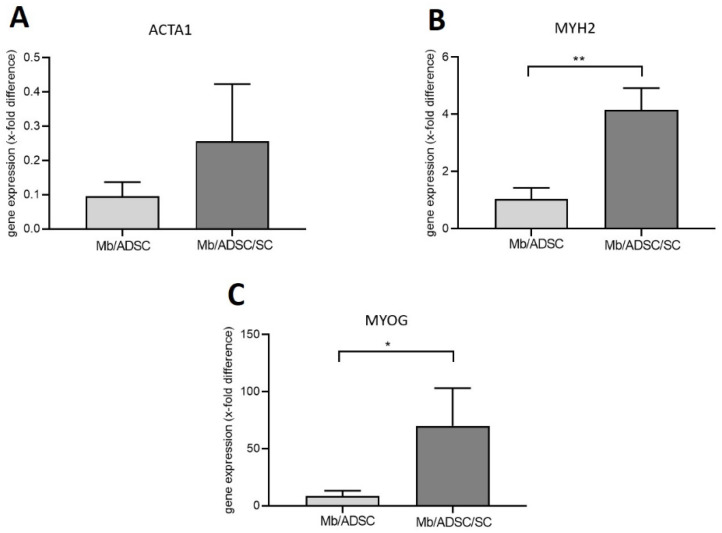
Gene expression of ACTA1 (**A**), MYH 2 (**B**), and MYOG (**C**) in Mb/ADSC vs. Mb/ADSC/SC after 28 days of myogenic differentiation on PCL-collagen I-nanoscaffolds. Expressions are demonstrated in x-fold difference compared with RNA from human muscle tissue using the 2^−ΔΔCt^ method. GAPDH was used as housekeeping gene in co-cultures and muscle tissue. MYOG and MYH2 were upregulated in Mb/ADSC/SC (69.97-fold ± 33.09-fold and 4.169-fold ± 0.756-fold, respectively) compared to Mb/ADSC (8.404-fold ± 4.8-fold and 1.038-fold ± 0.396-fold, respectively). * *p* < 0.05 (unpaired *t*-test), ** *p* < 0.001 (unpaired *t*-test).

Mb/ADSC or Mb/ADSC/SC were co-cultured on PCL-collagen I-nanoscaffolds and were myogenically differentiated for 28 days. Gene expression of MYH2 and MYOG was upregulated by addition of SC (*p* = 0.003 and *p* = 0.033, respectively). There was no difference in gene expression of ACTA1 between Mb/ADSC/SC (0.256-fold ± 0.167-fold) and Mb/ADSC (0.096-fold ± 0.041-fold) (*p* = 0.183) (Figure 9).

**Figure 10:**
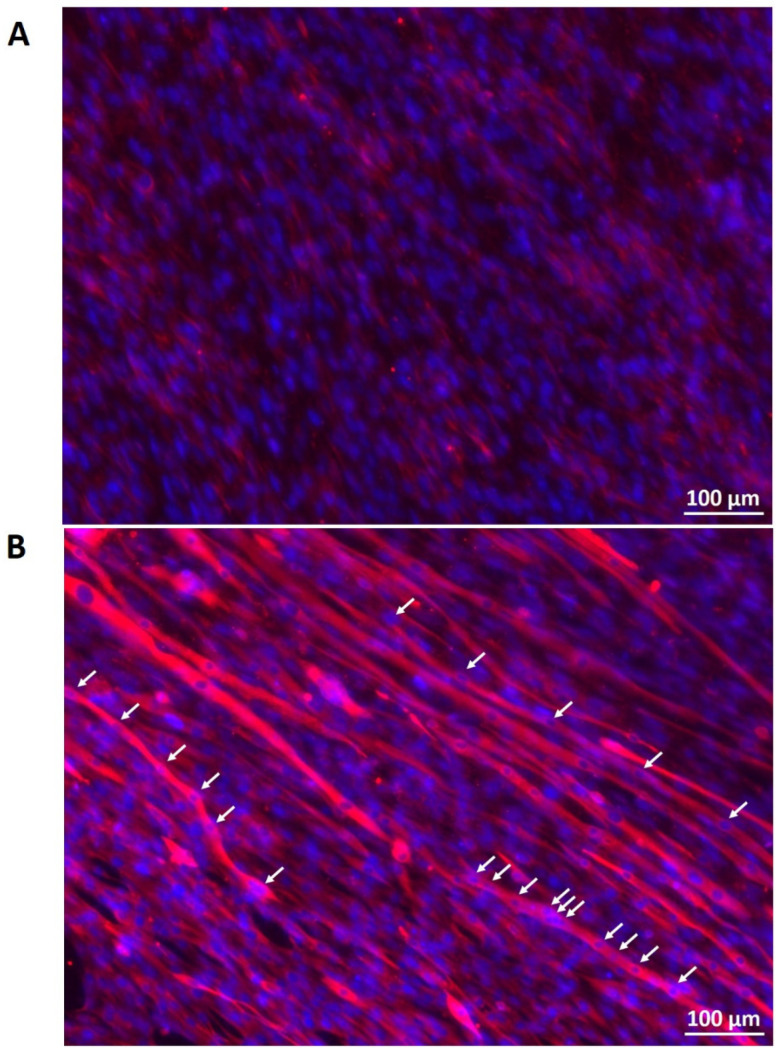
Myogenic differentiation and cell configuration after 28 days of myogenic differentiation of Mb/ADSC (**A**) and Mb/ADSC/SC (**B**) on PCL-collagen I-nanofibers. Fluorescence microcopy shows MHC positive (red) cells with clear evidence of highly aligned multinucleated (DAPI = blue) cells as possible myotube formation (white arrows point to nuclei in myotubes) in Mb/ADSC/SC. Scale bar 100 µm.

With fluorescence microscopy, the myogenic differentiation potential of Mb/ADSC vs. Mb/ADSC/SC seeded on PCL-collagen-nanoscaffolds was analyzed after 28 days of myogenic differentiation. Both groups expressed MHC. Only in Mb/ADSC/SC there was a clear evidence of highly aligned multinucleated cells as possible myotube formation (Figure 10).

## 4. Discussion

The findings of this study implicate that SCs enhance the myogenic differentiation of Mb/ADSC co-cultures in a 2D environment and on PCL-collagen I-nanoscaffolds. SCs led to higher gene expression of key myogenic markers and a higher grade of myotube maturation.

In contrast to a prior study, in which a long-term myogenic differentiation of Mb/ADSC on PCL-collagen I-nanoscaffolds under serum-free conditions was performed [12], the results of the current study demonstrate a significant increase of CK activity over time, in particular for the groups with a higher proportion of SCs. CK activity has been used as a biochemical marker for direct measurement of myogenic differentiation, at least in mouse myoblast cell lines [28,29,30]. Based on these results, we used a ratio of 1:1:0.5 Mb/ADSC/SC since a higher proportion of SCs (1:1:1) did not lead to higher CK activity.

In 2D co-cultures, no maturated myotubes could be observed in either group after 1 week of myogenic differentiation. This is in accordance to other studies where late myogenic markers were present after a time period of approximately 4 weeks, similar to the differentiation period in the present study [12,31,32].

The current study differs from our prior study in that primary human cells were used for the first time. Mbs were expanded to P5, which is a relatively high passage in comparison to the passages used in earlier studies [10,12]. This passage was used due to desmin positivity and fusion capacity after exposition to a myogenic differentiation medium. The addition of SCs to Mb/ADSC promoted myotube formation in the long term and enhanced the gene expression of key myogenic markers, especially MYOG and MYH2. Myogenin belongs to the myogenic regulatory factor family and plays a crucial role in directing myoblast fusion into multinucleated myofibers in the context of muscle regeneration [33]. The essential role of myogenin during muscle regeneration is further highlighted by the upregulation of its gene expression after facial nerve resection in rats, and the prevention of muscle atrophy following denervation [34]. Similarly, Cui et al. showed the upregulation of myogenin of Mbs by addition of SCs through ciliary neurotropic factor [19]. Myosin heavy chain is a motor protein that dictates contraction of the myofiber [33]. During myogenesis, maturation of the myofiber is corroborated by the expression of MHC and other contractile proteins [35]. It has been shown that co-culture of myoblasts with motor neurons maintained slow MHC expression [14] and enhanced the neonatal form of MHC [36]. We did not distinguish between different isoforms of MHC in our study since our interest focused on myogenic differentiation in general, rather than on the individual developmental steps.

Several studies have reported a positive effect of neuronal progenitor cells like embryonal motor neurons or neuronal slices from newborn rats on myotube formation and neuromuscular junction formation [14,15,16]. The use of these cell types limits translation into a clinical setting. Although Ostrovidov et al. demonstrated that the co-culture of PC12 neural cells and C2C12 myoblasts improved the levels of myogenic differentiation and myogenic gene expression in 3D hydrogels [35], these cell lines are also unsuitable in a clinical setting [37]. By using primary human cells, seeded onto a biocompatible matrix, we established a 3D model, which is structurally and physiologically similar to human muscle tissue, facilitating application in translational medicine. Recent studies have shown a regenerating effect of SCs on muscle tissue: both SCs and myotubes are regenerated via the same pathways, including the mTOR and Wnt signaling pathway [18,38]. SCs are known to secrete growth factors, amongst them ciliary neurotrophic factor, that regenerates peripheral nerves and accelerates the proliferation of myoblasts [19,38]. Cui et al. demonstrated that HA-19, an oleanolic acid derivative, increased the proliferation of SCs and subsequently led to weight gain of denervated muscles in a muscle atrophy mouse model [19]. Compared to the above mentioned references, this is the first study proving that SCs are suitable for creation of de novo muscle, given the enhanced myogenic differentiation of muscle progenitor cells in co-culture with MSCs.

As described previously, the PCL-collagen I-nanoscaffolds used in the present study represent a biocompatible and stable matrix for 3D tissue engineering of skeletal muscle and can be easily translated into a clinical application, since acetic acid is used as a benign solvent [13]. The matrices enabled the long-term differentiation of Mbs in co-culture with MSCs and parallel alignment on the nanofibers [12]. In the present study, a wst-8-assay and a live dead assay were performed to evaluate cell cytotoxicity of the scaffolds and to clarify cell status, because differences in cell viability could be a confounding factor for myogenic differentiation. The results demonstrated that there was no difference between the groups after myogenic differentiation at any period of time. Surprisingly, AI increased over time after initial decrease for Mb/ADSC/SC. This might be caused by the formation of more myotubes similarly to the Mb/ADSC/SC 2D co-cultures. The presence of multinuclear cells, undergoing apoptosis would lead to a higher AI compared to apoptotic unfused cells while number of nuclei remained unchanged. Thus, AI and NI might only be suitable for the evaluation of proliferating cells on PCL-collagen I-nanoscaffolds. Unfortunately, we did not quantify MHC-expression on PCL-collagen I-nanoscaffolds. 3D distribution of the fibers and of the cells seeded onto them leads to myotube formation in different layers on the scaffold, which inhibited taking clear immunofluorescence pictures of the whole scaffold and determining MFI and MMI. However, mature myotubes could be clearly demonstrated only in the Mb/ADSC/SC group.

There are several limitations of this study. First, the expression of neuromuscular junction markers, which are known to increase in nerve–muscle constructs, remains unknown in the present study since we focused on (late) myogenic differentiation markers. Second, we used commercially purchased primary SCs. The collection of human nerve-derived SCs might require invasive surgical resection, leading to donor site morbidity. One possible solution for this issue could be the isolation of SCs from skin, which has been successfully demonstrated by Stratton et al. In this study, adult human nerve and skin-derived SCs were genetically indistinguishable and secreted factors that promote neurite outgrowth and myelinate axons [39]. We did not analyze human Mb monocultures as a control group, as was carried out in our prior study of rat (co-)cultures [40]. Yet, the purpose of the present study was to compare the co-culture of Mbs and ADSCs, which was shown to be a valuable combination in terms of myogenic differentiation [40], to the co-culture of Mbs, ADSCs, and SCs. As Mb monocultures are not suitable for engineering large-scale muscle constructs for the reasons mentioned above, the combination of Mb, ADSC, and SC is a valuable option for that purpose.

Given the results of this study, we propose that the co-culture of Mb/ADSC and peripheral neural cells on PCL-collagen I-nanofibers serve as a platform, promoting myogenic differentiation. This model can be easily transferred into an in vivo model like the arterio-venous loop model of the rat to engineer vascularized large-volume constructs [41]. Modifying this model by integration of a motoric nerve might enable the creation of functional muscle tissue, which can later be transplanted into a defect site [40].

## 5. Conclusions

The findings of this study highlight that the presence of SCs improves myogenic differentiation of co-cultured primary human Mbs and ADSCs, enhancing myotube formation and the expression of myogenic key markers involved in muscle development and function. Our results show that neural communication not only has a fundamental impact on synaptic signaling but also on de novo muscle formation. The co-culture of Mbs, ADSCs, and SCs on electrospun PCL-collagen I-nanoscaffolds as a biocompatible matrix mimics the topographical and biological cues of skeletal muscle and enables later transplantation of the construct into an in vivo setting, to engineer vascularized and functional large-volume muscle constructs.

## Figures and Tables

**Table 1:** Primer sequences.

	Forward Primer	Reverse Primer
MYOG	TGCCATCCAGTACATCGAGC	TGTGAGAGCTGCATTCGCTG
MYH2	GGGCCTTTCAAGAGGGACAC	TGCGCTCCCTTTCAGACTTT
ACTA1	CACAATGTGCGACGAAGACG	CTCTCTTGCTCTGAGCCTCG
GAPDH	TCCACCCATGGCAAATTCCA	TTCCCGTTCTCAGCCTTGAC

## Data Availability

The data that support the findings of this study are available from the corresponding author upon reasonable request.

## Supplementary Materials

The following supporting information can be downloaded at: https://www.mdpi.com/article/10.3390/cells11091436/s1, Figure S1: Desmin staining of Mb of PP3 in P2-4 (left panel). Figure S2: FACS analysis of ADSC in P3 (left) and in P6 (right).
