# Supplementary material for: Schwann Cells Promote Myogenic Differentiation of Myoblasts and Adipogenic Mesenchymal Stromal Cells on Poly-ɛ-Caprolactone-Collagen I-Nanofibers
Source: Cells. 2022 Apr 24;11(9):1436. doi: 10.3390/cells11091436 (PMC9100029; doi:10.3390/cells11091436)
Supplement: Supplementary file 1 [file cells-11-01436-s001.zip › cells-1668748-supplementary.pdf]

Supplementary Material

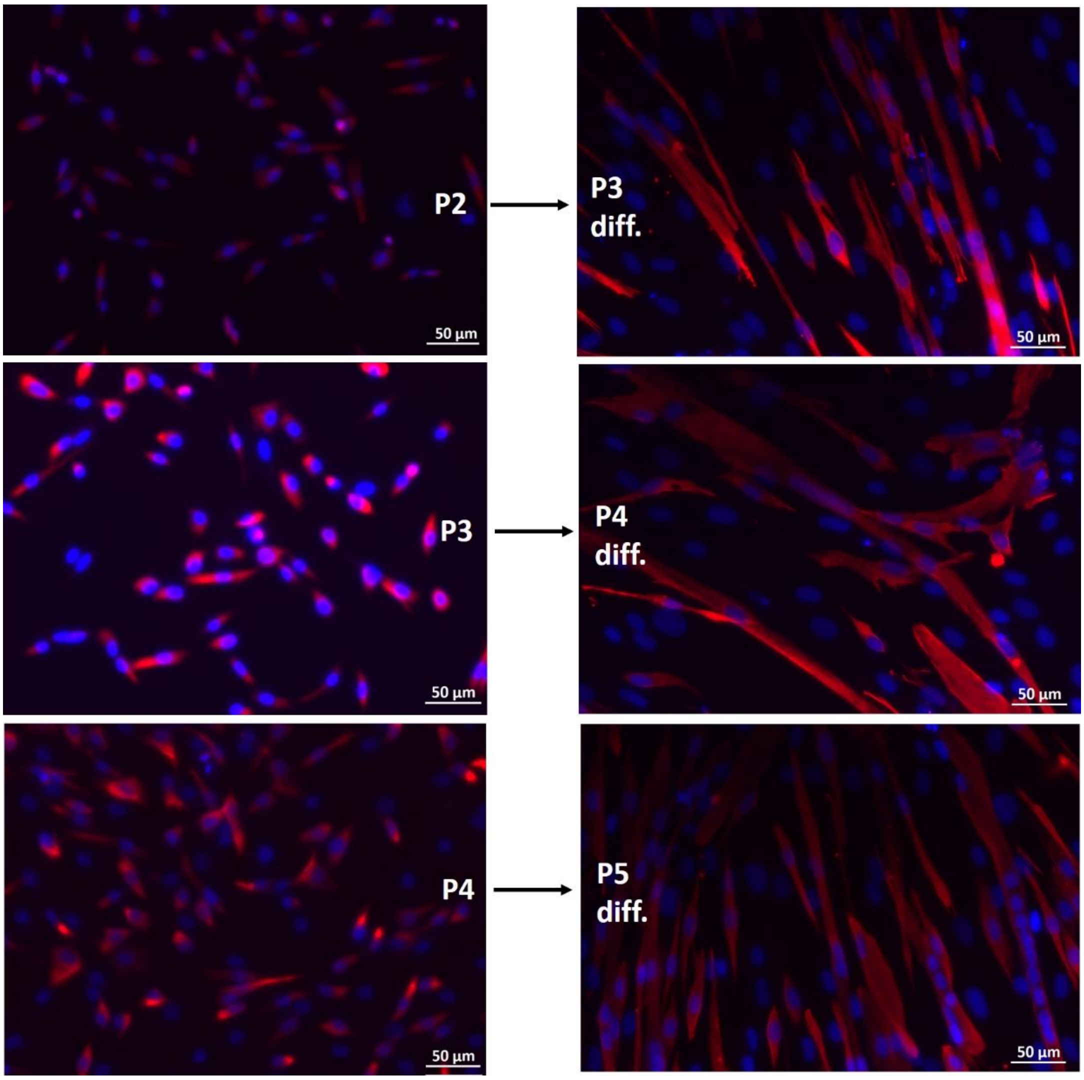

Figure S1: Desmin staining of Mb of PP3 in P2-4 (left panel). Mb of the corresponding passage were seeded and myogenically differentiated for 7 days (right panel). After seeding, Mb were referred to as one passage higher than the original passage.

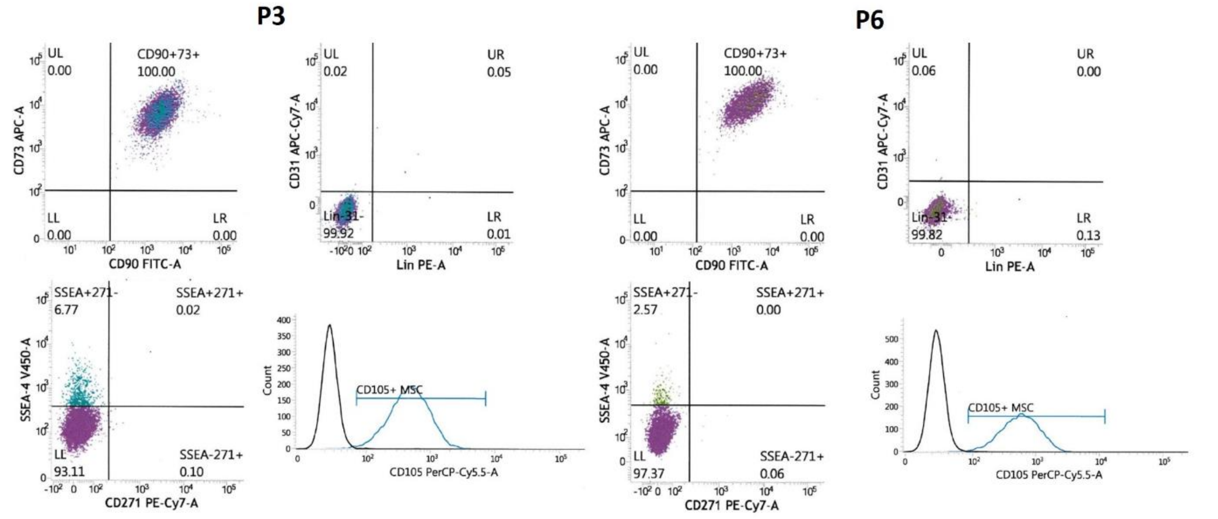

Figure S2: FACS analysis of ADSC in P3 (left) and in P6 (right). ADSC surface markers (CD90, CD73, CD105), negative markers (Lin = CD34, CD11b, CD19, CD45, HLA-DR; CD31; CD271), and SSEA-4 (stage-specific embryonic antigen-4) were determined.
